# Patient-Derived Midbrain Organoids to Explore the Molecular Basis of Parkinson's Disease

**DOI:** 10.3389/fneur.2020.01005

**Published:** 2020-09-04

**Authors:** Benjamin Galet, Hélène Cheval, Philippe Ravassard

**Affiliations:** Molecular Pathophysiology of Parkinson's Disease Group, Paris Brain Institute (ICM), INSERM U, CNRS UMR 7225, Sorbonne University, Paris, France

**Keywords:** Parkinson's disease, IPS (induced pluripotent stem) cell, organoid, midbrain, dopamine, genetics

## Abstract

Induced pluripotent stem cell-derived organoids offer an unprecedented access to complex human tissues that recapitulate features of architecture, composition and function of *in vivo* organs. In the context of Parkinson's Disease (PD), human midbrain organoids (hMO) are of significant interest, as they generate dopaminergic neurons expressing markers of *Substantia Nigra* identity, which are the most vulnerable to degeneration. Combined with genome editing approaches, hMO may thus constitute a valuable tool to dissect the genetic makeup of PD by revealing the effects of risk variants on pathological mechanisms in a representative cellular environment. Furthermore, the flexibility of organoid co-culture approaches may also enable the study of neuroinflammatory and neurovascular processes, as well as interactions with other brain regions that are also affected over the course of the disease. We here review existing protocols to generate hMO, how they have been used so far to model PD, address challenges inherent to organoid cultures, and discuss applicable strategies to dissect the molecular pathophysiology of the disease. Taken together, the research suggests that this technology represents a promising alternative to 2D *in vitro* models, which could significantly improve our understanding of PD and help accelerate therapeutic developments.

## Parkinson's Disease

### Pathophysiology

Parkinson's Disease (PD) is the second most frequent neurodegenerative disorder after Alzheimer's Disease. It affects over 10 million people worldwide, with an estimated yearly cost of 52 billion dollars in the United States alone, and an increasing prevalence due to an aging population (1). Although PD has historically been characterized by its motor symptoms (bradykinesia, tremor, and rigidity), the frequent co-occurrence of cognitive and psychiatric symptoms (such as apathy, depression, and executive dysfunction) have led to the revaluation of PD as a quintessential neuropsychiatric disorder (2).

At the cellular level, the central hallmark of PD is the misfolding and aggregation of α-synuclein (α-syn), a protein involved in neurotransmitter release, membrane remodeling and vesicle recycling, into toxic ß-sheet rich fibrillar aggregates (3). While impairments in protein synthesis, folding, and degradation have been extensively linked to α-syn aggregation and toxicity, recent advances have also highlighted the importance of lipid dysregulation in its pathological mechanisms (4). In most cases, these alterations lead to the accumulation of α-syn aggregates along with mis-trafficked lipid vesicles and organelles into inclusions termed Lewy Bodies (LB) and neurites (5, 6), which are considered to be the main histological manifestation of PD. While it remains unclear whether LB themselves play a harmful, protective or even “neutral bystander” role in PD (7), α-syn-associated pathology eventually induces the degeneration of vulnerable cells through altered mitochondrial, proteasomal, and autophagy-lysosomal pathways (6, 8).

This vulnerability has been linked to several cellular characteristics: elaborate neuronal arborization with many vesicular release sites (enriched in α-syn), elevated intracellular calcium concentrations due to autonomous pacemaker activity, and higher basal levels of mitochondrial oxidative stress (9). These characteristics are all found in the dopaminergic (DA) neurons of the *substantia nigra* (SN), which are the most affected neuronal type in PD. Their progressive degeneration leads to a massive loss of DA release within cortico-basal ganglia networks, and the emergence of both motor and psychiatric symptoms of PD (10). The symptomatology is further broadened by alterations of other neuronal types throughout the course of the disease, although to a lesser extent (11). These include other neuromodulator-producing neurons [cholinergic (12), noradrenergic (13), and possibly serotonergic (14, 15)], enteric neurons (16), as well as cortical neurons, in which comorbid Alzheimer's Disease pathology can arise in later stages (17, 18). Additionally, microglia and astrocyte-mediated neuroinflammatory processes are also known to contribute to neurodegeneration and the progression of synucleinopathy (19, 20).

PD is also a highly heterogeneous disease, as patients can present significant differences for example regarding age of onset (21), adherence to Braak staging (9), alteration in neurotransmitter systems (22–27) and symptom presentation (9, 21, 28, 29). This heterogeneity thus suggests that the etiology of PD may involve a diversity of molecular and cellular mechanisms, which remain to be fully identified.

### Molecular Basis

Much of our understanding of the pathological mechanisms of PD come from the study of relatively rare, high risk/monogenic forms of the disease. To this day, 19 disease-causing genes have been identified, amongst which 10 are autosomal dominant (including mutations in *SNCA*, which encodes α-syn, and in the *Leucine Rich Repeat Kinase 2* / *LRRK2* gene), and 9 autosomal recessive (including *PRKN, PINK1*) (30). PD cases due to mutations in those genes however only represent 5–10% of all cases. Interestingly, the G2019S LRRK2 mutation has a variable penetrance, as it can lead to both sporadic and familial PD (31).

The most recent GWAS meta-analysis to date has identified 90 common genetic variants with medium to low effect sizes that were associated with PD (32). This study also found that the expression of candidate genes was exclusively enriched in neuronal cell types (with the strongest enrichment residing in SN DA neurons, followed by pallidal, thalamic, and cortical neurons), a striking contrast with recent reports on the genetic architecture of Alzheimer's Disease which heavily implicated peripheral and CNS glial cell types (33) (blood, spleen, lung, and microglia). Gene ontology analyses also revealed enrichment for pathways referring to cellular stress responses and suggest a potential implication of neuro-inflammatory mechanisms. Interestingly, no significant association with other neuromodulator-producing neurons (serotonergic, noradrenergic, cholinergic) was revealed in these analyses, thus highlighting the centrality of DA and DA-associated networks in PD pathophysiology. This result may nevertheless be due to the fact that this study did not account for PD subtypes (21), which may be associated with alterations in different neurotransmitter systems (22–27). In this regard, future studies integrating large cohort GWAS data with patient stratification strategies may help identify molecular mechanisms driving PD heterogeneity.

Amongst the most highly significant and best characterized risk variants are those in the *beta-glucocerebrosidase* (*GBA*) gene. Such variants seem to impair lysosomal function and can lead to an increase in PD risk between 2- and 19-fold, and are associated with a more severe clinical profile regarding symptomatology and progression rate (21, 30). Interestingly, the presence of multiple risk variants in a single patient (referred to as the “polygenic load”), has also been shown to influence age of disease onset, but not the rate of progression (34).

Recent population studies have yielded PD heritability rates ranging between 0.22 and 0.27 (32, 35), suggesting that a majority of cases may be due to the interaction of genetic and environmental factors [rural living and pesticide exposure are well-known risk factors, while tobacco, coffee, and moderate alcohol consumption may be protective, see review (36)], and to stochastic processes. Mosaicism may for instance be a non-negligible contributor to the pathogenesis of sporadic PD (37), as changes in copy numbers of the *SCNA* gene have been observed in patient SN DA neurons (38). Nevertheless, only a minor fraction of the disease's heritability (16–36% depending on its prevalence) can be explained by the most recently identified risk loci (32), indicating that much of the “missing heritability” remains yet to be uncovered. This may be partly achieved through better understanding of epistatic interactions and the functional annotation of the non-coding genome, in which a majority of the single nucleotide polymorphisms (SNPs) fall. Indeed, like many other complex polygenic human diseases, the etiology of sporadic PD is likely attributable to the interactive effects of a high numbers of variants on the regulation of large-scale genetic networks (39). A growing body of research is for example revealing how non-coding variants affecting long range enhancer/promoter interactions or non-coding RNA may be involved in PD pathophysiology (40–43). However, as non-coding sequences tend to be less conserved between species, appropriate human models of the disease are thus required to expand our understanding of the molecular basis of PD.

## Modeling PD *in vitro*

### Reproducing Midbrain Development *in vitro*

Given the importance of DA degeneration in PD, human induced pluripotent stem cell (hiPSC)-derived DA cultures constitute highly relevant biological models to study the associated molecular mechanisms *in vitro*.

Midbrain DA (mDA) neurons are found in 3 separate nuclei: the *Substantia Nigra pars compacta* (SN, forming the A9 group), Ventral Tegmental Area (VTA, A10 group,) and Retrorubral Field (RRF, A8 group). A9 mDA neurons, which primarily project to sensorimotor and associative striatal areas (putamen and caudate nucleus), as well as some cortical areas, are particularly vulnerable to neurodegeneration in PD (10, 11).

In order to generate mDA neurons *in vitro*, several protocols have been established based on our understanding of midbrain development [see in depth reviews (44, 45)]. To summarize, stem cells are initially directed toward a neuroectodermal fate through TGFβ/activin/nodal and BMP pathways inhibition [referred to as dual SMAD inhibition (46)], using different combinations of molecules. SHH, WNT, and FGF8 signaling are then typically modulated in order to specify midbrain floor plate identity, from which mDA progenitors arise. Cells are then differentiated and matured through the use of neurotrophic factors such as brain and glial-derived neurotrophic factors (BDNF, GDNF) and Ascorbic Acid, a commonly used antioxidant. Correct specification should induce the expression of transcription factor (TF) FOXA1/2 in mDA progenitor cells, which in turn regulates the expression of LIM homeobox TFs LMX1A and LMX1B. These TFs are required for the specification and differentiation of mDA neurons, notably by up-regulating *NURR1, PITX3*, and *Tyrosine Hydroxylase* (*TH)*, which together constitute essential markers of mDA neuron identity. The differentiation and survival of mDA neurons is then regulated by EN1/2 homeobox genes, which remain expressed in adult neurons (44). It is worth noting that these differentiations protocols do not generate SN-like mDA neurons specifically, but rather a diversity of mDA subtypes (47), out of which some neurons express markers of A9 or A10 identity.

### hiPSC-Derived Models of PD in 2D

The development of these protocols triggered a wave of characterization studies aiming at identifying altered phenotypes of 2D mDA cultures derived from patient hiPSCs carrying monogenic (*PAKR2, PINK1, LRRK2, SNCA, GBA*, and *OPA1*) or sporadic forms of the disease. These phenotypic effects have been well-described in the literature, both at the cellular and molecular levels [see reviews (48–51)]. To summarize, several converging pathological mechanisms that contribute to the vulnerability of human mDA neurons were reproduced *in vitro*, including reductions in neuronal arborization, increases in α-syn expression, oxidative stress, and mitochondrial dysfunctions (decreased respiration and ATP production, impaired mitochondrial biogenesis), as well as altered cellular stress responses [such as the unfolded protein and integrated stress responses, which involve the endoplasmic reticulum (52–54)].

While these experiments helped validate hiPSC-derived mDA neurons as human cellular models of PD and achieve a better understanding of the cellular and molecular dysfunctions involved, only a few studies have however reported mDA degeneration (55, 56). Not surprisingly, the weeks-long differentiation of these DA neurons (up to 3 months) raises the limitations of these 2D cultures relatively to human development, in particular regarding neuronal maturity and the establishment of synaptic connections to other cell types. This has partially been taken into account using microfluidic devices that recreate direct contacts between mDA neurons and striatal medium spiny neurons (57), or using co-cultures with astrocytes (58). These approaches however do not allow the development of mDA neurons concomitantly with other cell types as it happens *in vivo*, which contributes to DA maturity and may be involved in PD mechanisms.

### Developing 3D Midbrain Organoids

In this context, the rise of human stem-cell derived brain 3D organoid cultures, which recapitulate features of the brain's composition, organization, and function (59), has led to significant advances in our understanding of neurodevelopment and in disease modeling. Although midbrain and mDA markers have been found to spontaneously arise in non-directed whole brain organoids (60), the proportions of cells expressing such markers tends to be small and highly variable, thus warranting the development of more directed differentiation protocols. While some approaches have led to the development of “neurospheres,” which contain an increased proportion of DA neurons (along with excitatory, inhibitory neurons as well as glial cells) (61), most efforts have been directed at specifically reproducing mesencephalic development in the dish, in order to generate mDA neurons in representative human “midbrain organoid” (hMO) structures.

**Figure 1:**
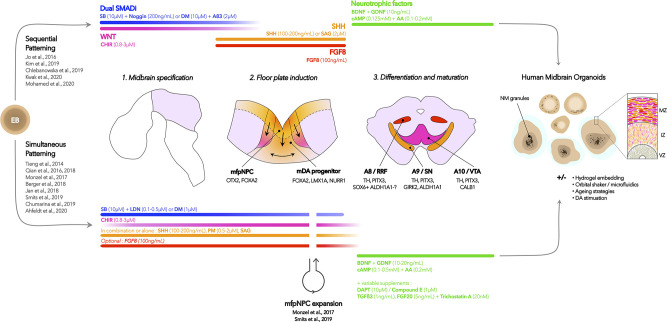
Summarized hMO differentiation strategies. hMO differentiation protocols aim at reproducing essential dynamics of *in vivo* human midbrain development, which are represented by the drawings in the middle section: (1) midbrain specification using dual SMAD inhibition and WNT modulation; (2) midbrain floor plate (mfp) induction through modulation of SHH and FGF8, and (3) differentiation and maturation of midbrain dopaminergic neurons using neurotrophic factors (see Table 1 for details in hMO models of PD). Each step is associated with the generation of cell types that can be identified *in vivo* and in hMO models using the mentioned markers. Starting from hiPSC-derived embryonic bodies (EB), the described protocols have either relied on the use of sequential or simultaneous patterning strategies, represented as the top and bottom branches, respectively. Optional modifications to the protocol include expansion of mfpNeural Progenitor Cells (mfpNPC), hydrogel embedding, use of orbital shakers or microfluidics devices, aging strategies, and DA treatment. The generated hMO typically contain mDA neurons expressing markers of A9 and A10 terminal differentiation, and long-term cultures may favor the apparition of neuromelanin granules, which can be enhanced through DA stimulation. Features of floor plate organization in ventricle, intermediate, and mantle zones (VS, IZ, MZ) may also be revealed using markers of mDA progenitors (65). This organization is particularly evident in hydrogel-embedded organoids, as this process favors apico-basal polarization. SB, SB431542; DM, dorsomorphin; CHIR, CHIR99021; A83, A-83-01; SHH, sonic hedgehog; SAG, smoothened agonist; FGF8: fibroblast derived growth factor 8; BDNF/GDNF, brain/glial-derived neurotrophic factor; AA, ascorbic acid; cAMP, cyclic AMP; LDN, LDN193189; PM, purmorphamine; TGFß3, Transforming growth factor beta 3; FGF20, fibroblast derived growth factor 20.

Tieng et al. (62) were the first to adapt a widely-used 2D differentiation protocol (63) to 3D suspension through the use of microwells to create homogeneously sized embryonic bodies, which were then placed on an orbital shaker for 3 weeks, before being seeded and grown at air-liquid interface. Although the suspension-culture phase of their protocol was short, they proved that such an approach could efficiently generate mDA progenitor cells (~80% of all cells expressed FOXA2 and LMX1A) as well as TH-expressing cells after only 3 weeks. Following these results, 3 new protocols were published within 1 year (64–66), describing the generation and long term maintenance of hMO (up to 5 months). These papers were the first to provide in depth characterization of the model, and proof that these organoids could be maintained in long term cultures in order to favor neuronal maturation. Although each protocol presents differences in timing, specific molecules used and their concentrations, these approaches mainly rely either on the sequential (65) or simultaneous (62, 64, 66) use of morphogens to induce midbrain floor plate identity, as described earlier (see Figure 1 for graphical summary). In order to promote nutrient and oxygen diffusion throughout the hMO, all of these initial protocols relied on the use of orbital shakers, as well as hydrogel embedding in some cases to promote apico-basal orientation and cellular proliferation.

These organoids developed features of organization similar to the midbrain floor plate, namely a ventricular zone containing OTX2+ FOXA2+ cells, as well as intermediate (LMX1A+ NURR1+) and mantle layers containing progressively maturing neurons (MAP2+ TH+). Several markers of pan-mDA neuronal identity have been consistently observed in hMO, including the dopamine transporter (*SLC6A3 /* DAT), DOPA decarboxylase enzyme DDC, and TF PITX3 (65, 67). While each study tried to estimate the proportions of mDA neurons in the hMO, differences in the methodologies and protocols used have led to variable results. For instance, by using FACS approaches, Jo et al. (65) found that at 2 months of differentiation, 22% of all cells were MAP2+ TH+, while Monzel et al. (66) found at the same timepoint a much higher yield of cells expressing essential markers of mDA identity: 61% were TH+FOXA2+LMX1A+. Nevertheless, both studies found that neuromelanin (NM) granules spontaneously appeared in long term cultures, their structures resembling those found in adult human SN tissue. Exogenous DA treatment could also significantly increase the accumulation of NM, suggesting that these granules may indeed be by-products of DA metabolism (65). While the authors did not try to dissect the diversity of mDA subtypes generated, which is in of itself a complex endeavor *in vivo* [see review (68)], NM-containing cells were indeed found to be enriched in transcripts expressed in A9 SN mDA neurons such as *KCNJ6 (GIRK2)* and *ALDH1A1* (47, 65, 69). Interestingly, ALDH1A1 may be particularly implicated in mDA neuron vulnerability to degeneration in PD (70). Some neurons were also found to be positive for CALB1, a marker of A10 VTA identity (65, 66). No study has however aimed at identifying A8 RRF-like neurons, likely due to the fact that they do not have a clear molecular signature (68). These organoids were also found to produce DA, and mDA neurons showed characteristic electrophysiological pacemaker activity which was responsive to the use of D2/D3 agonist quinpirole. Beyond mDA neurons and their progenitors, excitatory and inhibitory neurons (62, 65) were found in these hMO, as well as astrocytes and myelinating oligodendrocytes, consistent with the composition of the midbrain (65, 66).

**Table 1:** Summary of studies using hMO to model PD.

**References**	**Genetic risk variants**	**Cellular Stressors**	**Protocol**						**%TH+ cells** **(controls)**	**PD-related phenotype**	**Therapeutic approaches**
			**SMADi**	**WNT**	**SHH**	**FGF8**	**Maturation**	**Scaffolding,** **agitation**			
Jan et al. (79)	***SNCA*** A53T	/	DM (1 μM) SB (10 μM)	CHIR (3 μM)	PM (0.5–0.75 μM)	/	BDNF, GDNF (10 ng/mL) cAMP (500 μM) AA (200 μM) TGFß3 (1 ng/mL)	Matrigel + orbital shaker	/	**↑***eEF2K* mRNA, linked to mitochondrial stress	/
Kim et al. (77)	***LRRK2*** G2019S	AO removal, MPTP (200–500 μM)	*Unclear*	CHIR (3 μM)	SHH (100 ng/mL)	FGF8 (100 ng/mL)	BDNF, GDNF (20 ng/mL) AA (200 μM) *until day 45*	Matrigel + orbital shaker	60% (Day 60)	↓ mDA neuron mRNA markers, neurite length ↑α-syn, mitophagy & autophagy markers, MPTP sensitivity • Description of an “aging” strategy (–AO) for hMO • Identification of *TXNIP* as a mediator of LRRK2 pathology • Therapeutic strategies rescue elements of phenotype	LRRK inhibition & *TXNIP* knock-down
Smits et al. (73)	***LRRK2*** G2019S	/	LDN (250 nM) SB (10 μM)	CHIR (3–0.7 μM)	SAG (0.5 μM)	/	BDNF, GDNF (10 ng/mL) cAMP (500 μM) AA (200 μM) TGFß3 (1 ng/mL) DAPT (10 μM)	/	54% (Day 70)	↑ mDA progenitor cells ↓ Number and complexity of mDA neuons • Implication of genetic background	/
Chumarina et al. (75)	***POLG1*** Q11R	/	LDN (100 nM) SB (10 μM)	CHIR (0.8 μM)	SAG (1–2 μM) SHH (200 ng/mL)	FGF8 (100 ng/mL)	BDNF, GDNF (10 ng/mL) cAMP (500 μM) AA (200 μM) TGFß3 (1 ng/mL) *DA (50 μM) start. day 30*	/	40% (Day 100)	↑ Number of mDA and DA-induced NM accumulation↑ Neuronal reliance on glycolysis • No alterations in mitochondrial function	/
Ahfeldt et al. (74)	***PRKN***^−/−^***DJ1***^−/−^***ATP***^−/−^	/	LDN (100 nM) SB (10 μM)	CHIR(1 μM)	SAG (1 μM) PM (2 μM)	/	BDNF, GDNF (10 ng/mL) cAMP (100 μM) AA (200 μM) DAPT (10 μM)	SpinQagitation	40% (Day 35)	• Different molecular & cellular phenotypes / mutation • Dysregulation of autophagy-lysosomal pathways in all lines↑ Mitochondrial stress, SNCA expression, in *PRKN*^−/−^↑ early death of mDA neurons in *PRKN*^−/−^ (A9 specific?)	/
Kwak et al. (67)	/	MPTP (10–100 μM)	Best: DM (2 μM) A83 (2 μM)	CHIR (Best: 3 μM)	SAG (2 μM)	FGF8 (100 ng/mL)	BDNF, GDNF (10 ng/mL) cAMP (125 μM) AA (200 μM) *DA (50 μM) start. week 8*	Matrigel (+I/L) + orbital shaker	86% of neurons (Day 35)	↑ Vulnerability of mDA neurons to MPTP toxicity	/
Chlebanowska et al. (82)	**Sporadic PD**	/	SB (10 μM) Noggin (200 ng/mL)	CHIR (0.8 μM)	SHH (100 ng/mL)	FGF8 (100 ng/mL)	BDNF, GDNF (10 ng/mL) cAMP (125 μM) AA (100 μM)	Matrigel (+I/L) + orbital shaker	/	↓*TH* expression↑*Pentraxin 3* (*PTX3*) expression	/
Monzel et al. (83)	/	6OHDA (50–500 μM)	DM (1 μM) SB (10 μM)	CHIR (3 μM)	PM (0.5–0.75 μM)	/	BDNF, GDNF (10 ng/mL) cAMP (0.5 mM) AA (200 μM) TGFß3 (1 ng/mL)	Matrigel + orbital shaker	~50% (Day 42)	↑ Vulnerability of mDA neurons to 6OHDA toxicity	/
Jarazo et al. (80)	***PINK1*** Q456X/I368N ***PRKN*** R275W	/	DM (1 μM) SB (10 μM)	CHIR (3 μM)	PM (0.5–0.75 μM)	/	BDNF, GDNF (10 ng/mL) cAMP (500 μM) AA (200 μM) TGFß3 (1 ng/mL)	Matrigel + orbital shaker	~45% (Day 30)	↓ Number of mDA neurons in *PINK1* hMO • HP-β-CD treatment increasesmDA neuron counts in *PINK1* and *PRKN* hMO	HP-β-CD

*DM, dorsomorphin; SB, SB431542; CHIR; CHIR99021; PM, purmorphamine; BDNF/GDNF, brain/glial-derived neurotrophic factor; AA, ascorbic acid; cAMP, cyclic AMP; LDN, LDN193189; SAG, smoothened agonist; I/L, short treatment with insulin (2.5 μL/mL) and laminin (200 ng/mL), mDA neuron, midbrain dopamine neuron; NM, neuromelanin*.

hMO hold a number of advantages over their 2D counterparts. RNA sequencing of hMO for instance showed that their transcriptomic profile was closer to that of prenatal midbrain samples compared to 2D cultures (63), with higher expression of mDA markers such as *ALDH1A1* and *KCNJ6 (GIRK2)*, as well as glial markers *OLIG3* and *SLC1A3 (EAAT1/GLAST)*. Interestingly, markers of non-dopaminergic catecholaminergic neurons such as *DBH* and *SLC6A2 (NET)*, which frequently arise in 2D cultures, were also found to be significantly decreased in hMO, thus highlighting the importance of the 3D environment for proper mDA specification. Tieng and colleagues (62) also showed that mDA neurons derived from 3D cultures expressed higher levels of *TH* and presented varicose-like neurites reminiscent of A9 neuronal morphology, which had not previously been observed in 2D cultures. Furthermore, the spontaneous or dopamine-induced apparition of neuromelanin granules (65–67) is a remarkable feature as it has rarely been found in 2D cultures (55, 71).

More recent evolutions of these protocols have confirmed the initial observations, as well as aimed at increasing the quality and reproducibility of hMO (67, 72–76) and better estimating their yield of mDA neurons. For instance, using a high content image analysis approach, Smits et al. (73) showed that TH+ cells composed 62% of all cells after 1 month of differentiation, while Ahfeldt et al. (74) found using a knock-in TH:tdtomato line, that TH+ cells composed ~38% of total cells at a similar timepoint. Such differences are likely to arise from cell line effects as well as protocol variations. Kwak et al. (67) recently aimed at establishing ideal conditions to maximize mDA neuron generation in hMO, by testing out different combinations of molecules for SMAD inhibition and modulating WNT signaling. These modifications allowed them to approximately double their yield of TH+ cells compared to commonly used molecule combinations (86% TH+ cells by day 28), and to efficiently suppress cortical marker expression. By 4 months of culture, their hMO were also producing higher concentrations of DA than previously reported. Taken together, these findings support the relevance of hMO cultures to obtain mDA neurons expressing markers of terminal differentiation (such as NM production) in a 3D environment that reproduces the neuronal and glial composition of the human midbrain.

### Midbrain Organoid Models of PD

The first two in-depth reports of PD modeling in hMO focused on the effects of the *LRRK2* G2019S mutation, which has been associated with both sporadic and familial forms of the disease due to its variable penetrance (31), and which constitutes the most common genetic risk factor for PD. To do so, the researchers relied on Crispr-Cas9 gene editing to either introduce the mutation in a control hiPSC line (77), or to combine this with a correction in a mutant patient line (73). Smits et al. (73) found that while the number of mDA progenitors (FOXA2+TH- cells) was significantly increased after 1 month of differentiation in *LRRK2* vs. control hMO, an apparent impairment of differentiation led to a reduction in the number and complexity of mDA neurons (FOXA2+TH+) after longer periods of culture (day 70). Interestingly, the increase in the number of progenitors was significantly higher in *LRRK2* PD hMO compared to those from controls with the knock-in mutation. This result thus highlights the importance of the genetic background in the penetrance of the *LRRK2* G2019S variant (31). In line with these findings, Kim et al. (77) observed that while *LRRK2* G2019S hMO were no different in size compared to controls, mDA neurite length and expression of mDA identity markers were decreased (such as *TH, DAT, NURR1, PITX3, EN1*) by day 60. The *LRKK2* hMO also contained higher levels of phosphorylated α-syn in endosomal compartments, and higher expression levels of markers of mitophagy and autophagy. The authors also identified TXNIP [a thiol-oxidoreductase that induces lysosomal dysfunction and DA cell death when overexpressed (78)] as an important mediator of *LRRK2*-G2019S pathological mechanisms, and proved that knocking-down its expression reversed the accumulation of phosphorylated α-syn.

More recently, an extensive report from Ahfeldt et al. (74) used hMO to study the roles of 3 severe PD-associated mutations (in *PRKN/PARK2, DJ1/PARK7*, and *ATP13A2/PARK9*) through genomic editing of a healthy control hiPSC line. RNAseq analyses of TH+ cells after 1 month of differentiation found that *PRKN*–/– mDA neurons showed the highest amount of differentially expressed genes (1641) compared to controls. While proteomics analyses revealed a dysregulation of the autophagy-lysosomal pathway in all cell lines, the *PRKN*–/– mDA neurons also showed an upregulation of pathways associated with oxidative phosphorylation, mitochondrial dysfunction, and Sirtuin signaling, as well as a significant depletion of mitochondrial proteins. Supporting these results, they found a significantly higher level of mitochondrial reactive oxygen species (ROS) in TH+ cells from *PRKN*^−/−^ hMO compared to their TH- counterparts and to control cells (both TH+ and TH–). Furthermore, while the mDA neuronal population was significant reduced in *PRKN*^−/−^ organoids (from 40 to 17% of all cells), there were no significant differences in the other two cell lines. The authors showed that this deficit was not due to an impairment in mDA generation, but rather to the death of newly differentiated TH+ neurons, which could be linked to a 3-fold increase in SNCA protein expression in these hMO. Interestingly, the expression of VTA marker CALB1 was 4x higher in the *PRKN*^−/−^ hMO, suggesting that A9-like neurons may have been more severely affected by the early neuronal death, thus leading to a bias in subtype generation. It is however not known if other mutations would have provoked a similar phenotype at later timepoints, although mDA neurons in *DJ1*^−/−^ and *ATP13A2*^−/−^ hMO also tended to show increases in mitochondrial ROS.

Reports of decreased mDA identity and impairment of mitochondrial function were supported by two additional studies which partly relied on hMO. For instance, *SNCA* A53T-mutated hMO recapitulated the increased expression of *eEF2K* mRNA found in *post-mortem* patient SN (79). eEF2K, also known as Calmodulin-dependent protein Kinase III (CamKIII), is a crucial regulator of protein synthesis and synaptic plasticity, and is involved in a-syn mediated mitochondrial toxicity (79). Mutations in *PINK1*, which encodes a mitochondrial kinase, have also been linked to reduced TH+ counts in hMO (80). Taken together, these studies suggest that hMO constitute a valid translational model to investigate the effects of different PD-associated mutations, as they reproduce elements of cellular pathology involving oxidative stress found in *post-mortem* tissue (81) (see Table 1 for summary).

Interestingly, a recent study focusing on a novel variation in the *POLG1* gene (Q811R), previously linked to progressive external ophthalmoplegia and parkinsonism (75), found significant increases in hMO TH+ cells after 100 days of culture compared to those from a gender-matched control. This study also reported an increased production of NM in response to DA treatment, which may have neurotoxic effects in the long run. Although no deficits in mitochondrial respiration were observed, metabolic and proteomics data indicated an increased level of glycolysis, which was specific to neurons. The striking differences from previously mentioned results (reduced mDA neuron counts and impaired mitochondrial respiration) indicate that *POLG1*-related PD may thus entail different pathological mechanisms. However, as isogenic lines were not used as controls in this study, the experiments should be replicated to confirm these findings.

hMO may also be of use to study sporadic forms of PD, including the effects of PD-associated environmental stressors. So far, only one study has aimed at deriving hMO from patients with sporadic PD (82). The authors found, in line with previous articles, a decrease in *TH* expression after 1 month of culture of hMO derived from 2 sporadic patients, compared to those from 2 healthy controls. This effect might have been linked to early decreases in *FOXA2* and *LMX1A* expression. They however also measured an increase in the expression of *PTX3*, which encodes a protein (Pentraxin 3) involved in neuroinflammatory responses that is increased in the plasma of PD patients (84). Nevertheless, as hiPSC-based studies of sporadic diseases are hard to control for, additional studies with increased statistical power are needed to further explore sporadic PD mechanisms in hMO. Sporadic PD dynamics may also be probed through exposure to mitochondrial stressors such a rotenone and MPTP, which have been shown to preferentially affect mDA neurons in hMO and related cultures (67, 77, 85).

As discussed earlier, the 3D nature of hMO favors better modeling of the *in vivo* midbrain over 2D cultures, and may by extension provide a better translational value when studying neurodegenerative disorders such as PD. A study for instance showed that in plated cultures of *LRRK2* G2019S mDA neurons, most of the PD phenotype (such as a reduction of the number and arborisation complexity of TH+ cells, impaired mitochondrial function and increased apoptosis) appeared only when Matrigel was used to recreate a 3D environment (86). Similarly, when comparing the transcriptome of hMO to 2D cultures of *LRRK2* G2019S-derived mDA neurons, Kim et al. (77) found that the genes differentially expressed in hMO were enriched for transcripts found in *post-mortem* PD tissue. In support of this finding, the expression of *TXNIP*, which they proved to be central to pathophysiological mechanism in *LRRK2* G2019S, showed 4-fold higher expression in hMO compared to 2D cultures. Altogether, these studies indicate that 3D hMO cultures may constitute a significant improvement over 2D cultures as *in vitro* platforms to model PD.

## Future Technological Challenges

### Challenges Inherent to Organoid Culture

It is somewhat surprising that several essential features of PD pathophysiology can be modeled in relatively young stem-cell derived structures, which may conceptually be better suited to study pathologies with clearly recognized neurodevelopmental components such as autism spectrum disorder (ASD), schizophrenia, lissencephaly, and many others (87). Furthermore, the reprogramming of differentiated patient cells to iPSC-states is known to have a “rejuvenating” effect by erasing many crucial aging-related epigenetic marks (88). Brain organoids have however also proven to be able to reproduce strong aging-related cellular phenotypes of Alzheimer's Disease (AD) (89–91). As tracking the earliest stages of PD or AD is an inherently difficult task, these results thus support the possibility that important neurodevelopmental aspects of such diseases may have been overlooked [see reviews (92, 93)].

However, another complementary possibility is that these severe phenotypes may partly be a by-product of organoid culture limitations. Indeed, although 3D organoid models constitute significant advances compared to their 2D counterparts, their density and size restrain the proper diffusion of oxygen and nutrients to all cells, leading to a well-known necrotic core. Brain organoids are also characterized by an upregulated reliance on glycolysis and high levels of ER stress, which may impair neuronal differentiation and promote mitochondrial stress (94), an aggravating factor in the context of neurodegeneration. Furthermore, while glial cells play an essential role of clearance in disorders such as PD and AD [see reviews (95, 96)], gliogenesis mainly happens in later stages of organoid culture (after 6 months in forebrain organoids) (97), and typically does not include microglial cells, unless differentiation protocols favor their apparition (98). In this sense, the stressful culture conditions and incomplete glial support may trigger and/or speed up pathophysiological cascades primed by genetic risk variants in PD hMO, and lead to the early apparition of severe neurodegeneration-related phenotypes.

#### Reducing *in vitro* Culture Artifacts

An essential endeavor to answer these questions will be to develop strategies to reduce culture-related artifacts, and to modulate cellular maturation in order to study early and later stage neurons and glia. Several studies have already started addressing these issues. For instance, it is now clear that transplantation inside rodent brains can effectively vascularise the organoids, correct artifacts linked to *in vitro* culture and significantly enhance neuronal maturation (94, 99, 100). These improvements nevertheless come at the expense of uncontrolled interactions between the host and grafted tissue, and synaptic integration into the host brain (100). It is however for now not known what effects such transplantations approaches would have on neurodegeneration-related phenotypes in organoid grafts.

Animal-free approaches may however also be of use. Biophysics studies of allometric scaling have for instance highlighted the importance of culture medium, hydrogel composition and microfluidic device uses in the context of 3D cultures (101–105). First of all, the composition of commonly used media for hMO culture should be scrutinized. Indeed, such composition may be partly responsible for the cellular stress and differentiation defects observed in several brain organoid cultures (94), as the abnormally high levels of glucose used in the vast majority of hMO protocols are known to impair the normal metabolic reprogramming of neural progenitor cells to neurons (106) through increased oxidative and ER stress (107). Furthermore, recent reports suggest that using culture medium with more physiological levels of glucose may be more adapted for neuronal maturation and modeling of neurological disorders (108–110). Secondly, the development of synthetic hydrogels as alternatives to animal-derived Matrigel and Geltrex may provide enhanced control and reproducibility of the 3D environment in which organoids grow (105). Thirdly, in order to compensate for diffusion limitations in organoids, two approaches have been described. Cakir et al. (111) for instance showed that cortical organoids genetically engineered to express *hETV2*, which encodes a transcription factor involved in endothelial differentiation, spontaneously formed a vascular-like network *in vitro* which dramatically reduced markers of cell death and hypoxia without the need for transplantation. Alternatively, microfluidic devices may also help increase oxygen and nutrient diffusion throughout the organoids, as evidenced in hMO cultures (112). Finally, electromagnetic stimulation may also be of interest to enhance neuronal differentiation (113, 114), including in hMO (115).

#### Addressing Variability in Organoid Differentiations

Organoid cultures have also gained notoriety for being highly variable, which can be a major issue for disease modeling and testing therapeutic approaches. This variability can be traced down to several crucial factors: differences due to the heterogenous genetic backgrounds of hiPSC cell lines, variations in hiPSC culture and differentiation protocols used, as well as batch effects. Nevertheless, each of these aspects may be addressed in order to improve the reproducibility of the model.

For instance, regarding the variability imputable to genetic background heterogeneity, several approaches may be adopted. The most straightforward path when studying variants carrying a high risk and penetrance is to generate isogenic controls using Crispr-Cas9 gene editing. Alternatively, in order to study lower-risk variants with reduced penetrance, more elaborate strategies may be necessary, such as relying on hiPSC lines from related donors, or taking into account polygenic risk scores in patient and control selection criteria in order to recreate a continuous variable for risk scores (116), an approach that has seen recent applications in the field of schizophrenia research (117).

Furthermore, the way in which hiPSC cells are cultured in the lab may have an important effect on their ability to generate reproducible organoid structures. Indeed, a recent study from Watanabe et al. (118) revealed that commonly used feeder-free hiPSC culture conditions (compared to fibroblast-supported), reduced their ability to generate reproducible high-quality cortical organoids by altering their pluripotency state. The authors however showed that these defects could be alleviated through the use of TGFß superfamily agonists, which increase the quality of organoid differentiation toward different brain areas as well as reproducibility across cell lines.

Importantly, the variability in brain organoid cultures was initially identified in whole-brain organoids, which rely on very little to no exogenous patterning, and which are very sensitive to cell line and batch effects (60). Several studies have since shown that this variability could be significantly reduced through the use of cytokines to guide and restrict organoid differentiation toward a specific regional fate. While this was initially demonstrated in forebrain organoid protocols (97, 119), a recent study from Nickels and colleagues (76) showed that hMO protocol refinement could also significantly reduce cell line and batch variability. Taken together, these approaches can thus help cut back on multiple sources of variability in hMO generation and improve their translational value for PD modeling and therapeutic discovery.

#### Aging in a Dish

As aging is the main risk factor for PD (120), understanding its mechanisms and reproducing them *in vitro* may also help build better disease models. At the molecular level, aging is associated with changes affecting the DNA's structure, content (reduced telomere length and mitochondrial copy numbers, increased DNA damage), epigenetic modulation (methylation clocks can reliably predict chronological age), and has identifiable transcriptomic, proteomic and metabolomic signatures [see reviews (121, 122)]. At the cellular level, aging is also characterized by a progressive accumulation of oxidative stress and mitochondrial dysfunction, a global increase in the number of cells baring features of senescence, as well as chronic low-grade inflammation (122). In the context of PD, both molecular (123, 124) and cellular dynamics (9) of aging have been identified as altered.

In this regard, perhaps the most problematic limitation of hMO as model systems for neurodegenerative diseases is that they rely on the use of cellular reprogramming, which has a rejuvenating effect on these molecular and cellular processes (88). Studies of epigenetics, transcriptomics, have for instance shown that organoids reproduce fetal molecular signatures of the human brain (125, 126). While this limitation does not prevent the study of disease-associated molecular aging mechanisms using hiPSC-derived cultures—a recent report showed that retinal organoids derived from Down Syndrome patients had a faster rate of DNA aging compared to controls (127)—it is a major obstacle to study aged states *in vitro*.

In order to bypass this limitation and reproduce aging phenotypes in a dish, several types of approaches have so far been described. Vera et al. showed that manipulation of telomere length, one of the molecular hallmarks of aging, through telomerase inhibition could induce other markers of aging (increased expression of γH2AX, a common marker of DNA damage, and increased oxidative stress) and accentuate PD phenotypes in hiPSC-derived 2D mDA cultures (128). A previous report from that group also found that a similar effect could be induced through overexpression of Progerin, the abnormal protein responsible for premature aging in Progeria syndrome (55).

Another more indirect alternative is to rely on the induction of cellular states associated with aging, for example through manipulation of oxidative stress *in vitro* with toxins (such as 6-OHDA, MPTP) or through changes in media formulation. For instance, in their 2019 paper, Kim et al. (77) removed antioxidants from the hMO culture medium after 45 days of culture (hMO-AO). While they did not provide a comparison with hMO+AO, they found that after 2 months of differentiation over a third of cells contained NM granules [compared to 7% at 146 days using a similar protocol with AO (65)], two thirds expressed markers of mature A9 neurons such as *GIRK2*, and over 40% expressed high levels of DNA damage (γH2AX+). Transcriptomics analyses also showed that control hMO-AO showed enrichment for “aged” human midbrain transcripts, and genes differentially expressed in *LRRK2*-G2019S hMO-AO were enriched for transcripts found in *post-mortem* PD midbrain tissue, thus supporting the relevance of their aging strategy. In this context, the basal level of ER stress characterizing organoid cultures (94) may also in of itself constitute an indirect aging strategy through alterations of global homeostatic mechanisms, including calcium homeostasis (129).

Finally, direct reprogramming of somatic cells such as fibroblasts into neural lineage cells (iNeurons, or iN) through transgenic expression of transcription factors (such as ASCL1 and NEUROG2), non-coding RNA, or even using small molecule cocktails, may constitute the most elegant way of inducing aging-related processes *in vitro*. Indeed, “aged” iNs preserve multi-level marks (epigenetic, genomic, transcriptomic, and proteomic) of aging and environmental interactions (130). This approach allows iN to maintain phenotypes such as defective mitochondrial function compared to hiPSC-derived neurons (131). While mDA neurons have already been generated using this approach (132), adapting this technology to hMO generation may however prove to be challenging, as published protocols for now rely on the direct conversion to post-mitotic neuronal types.

#### Enhanced Organoid Designs

**Figure 2:**
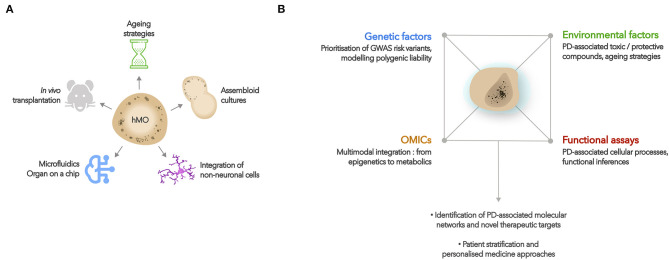
Developments and applications of hMO cultures. **(A)** Potential developments of hMO cultures include aging strategies, fusions with other brain region organoids, co-cultures with non-neuronal lineage cells (such as microglia, endothelial cells), use of microfluidics or “organ on a chip” approaches, and *in vivo* transplantations. **(B)** hMO constitute relevant biological platforms to study the effects of PD-associated genetic and environmental factors on cellular function and molecular networks. Such approaches may lead to a better understanding of the molecular basis of PD, help identify new therapeutic targets, and develop personalized medicine approaches.

The use of hMO can also be expanded by taking advantage of the flexibility of organoid cultures (see Figure 2A for graphical summary). For instance, organoids can be completed with non-neuronal lineage cells that do not typically arise during neural organoid differentiations, but which may be of interest for disease modeling. In the context of PD, co-culturing hMO with microglia-like and endothelial cells could for example enable researchers to study neuroinflammatory mechanisms involving glial activation and brain-blood-barrier disruption (133).

Several teams have indeed shown that hiPSC-derived microglia-like cells (iMG) (134–136) as well as immortalized human microglia (137) could efficiently colonize organoids when cultured together. These integrated microglial cells develop extensive ramified branching, and respond to challenges such as physical injury, stimulation with lipopolysaccharides, corticosteroids and amyloid-β-42 (amyloid-β-42) oligomers, as well as infection with Zika and Dengue viruses (135, 137–139). Two studies in particular showcase how iMG-organoid co-cultures may be of use to model neurodegenerative diseases. First of all, Lin et al. (89) showed that iMG carrying an *APOE4* genotype (a high AD risk allele of the *APOE* gene) had an altered morphology and reduced ability to clear extracellular Aβ aggregates in organoid co-cultures compared to their (low-risk) isogenic *APOE3* counterparts. Secondly, a study from Song et al. (139) proved that iMG were sensitive to the regional identity of the brain organoids they integrated (in this study, dorsal vs. ventral forebrain), and that this microenvironment impacted their response to Aβ-42 stimulation. Given that microglia play an important role in PD pathophysiology (96) and are influenced by regional specificities (140, 141), such co-cultures approaches may thus constitute a relevant strategy to study neuroinflammatory interactions.

Furthermore, as alterations in blood-brain barrier (BBB) function contribute to neuroinflammatory processes in PD, assessing the interaction between endothelial cells (ECs), pericytes and hMO may also be of interest. While this may be partly achieved through *in vivo* transplantation in rodents, the fact that the vascularization originates from the host (99, 100) may be a considerable limitation to study pathological cellular interactions. More elaborate strategies can however help overcome this issue. For instance, the transgenic induction of *hETV2* expression in organoids mentioned earlier (111) leads to the formation of a vascular structure reproducing key elements of BBB identity and function *in vitro*, which is sensitive to the disrupting effects of Aβ-42 oligomers. As an alternative, a vascular system can also be initiated *in vitro* by co-culturing organoids with hiPSC- or human umbilical vein-derived ECs, before proceeding to transplantations (142, 143).

Nevertheless, despite mDA neuron neurodegeneration being the central element of PD pathology, there is also evidence of a loss of cholinergic, adrenergic, and potentially serotonergic neurons over the course of the disease, which alters cortical and basal ganglia function and has been linked to several non-motor symptoms (11–15). Moreover, cortical regions can also be affected by Amyloid-ß and Tau pathology, which are associated with PD dementia (17, 18). In this context, using organoids differentiated toward different brain regions can help address the extended PD picture. Newly characterized brainstem organoids are particularly relevant as their composition encompasses midbrain and hindbrain structures, in which arise not only mDA, but also serotoninergic, cholinergic, and noradrenergic neurons (144). Cortical, subpallial, and thalamic organoids have also been well-characterized (145), and may be studied independently or fused with hMO/brainstem organoids to recreate elements of basal ganglia circuitry involved in PD (146–148). Such structures, named “assembloids,” could thus be used to study cellular interactions and molecular phenotypes in interconnected structures and to address more complex questions *in vitro*. For instance, what are the effects of genetic and environmental risk factors on different neuromodulator-producing cell types and their connectivity to forebrain structures? Why are striatal cells, despite receiving massive inputs from the SN, seemingly less vulnerable to synucleinopathy compared to cortical neurons (9)?

Finally, beyond the central nervous system (CNS), there is increasing evidence for an important role of the enteric nervous system (ENS) in PD pathophysiology (16), which may be addressed using intestinal or engineered ENS organoids (149). In this regard, a first study comparing the transcriptomic profiles of intestinal and neural organoids derived from *LRRK2* G2019S patient hiPSCs to those from healthy controls reported a wide range of alterations in biological processes and pathways in both models, suggesting that this path should be further explored (150).

### Tools to Explore the Molecular Basis of PD Using hMO

While hMO have for now mainly been used to study the effects of high-risk variants on cellular and molecular phenotypes, combining the access to human tissue provided by organoids with GWAS and -OMICs data provides an unbiased approach to further explore the genetic networks, cell types and developmental stages implicated in PD pathophysiology (see Figure 2B for graphical summary).

For instance, while GWAS data is often integrated with expression quantitative trait loci (eQTL) and *post-mortem* data to predict candidate risk genes with some tissue specificity, recently developed approaches may help researchers extract additional relevant information. For example, H-MAGMA (Hi-C-coupled MAGMA) can further improve candidate gene identification by incorporating chromatin interaction profiles from human brain tissue across neurodevelopmental stages (151). Cell-type specificity may also be explored more finely by integrating GWAS data with single-cell RNA sequencing (scRNAseq) datasets from the target tissue. Such an approach recently allowed Bryois et al. (152) to reveal a significant association of PD with cholinergic, monoaminergic, and enteric neurons as well-oligodendrocytes using scRNAseq data from a whole CNS. Although the main findings of this study were replicated in *post-mortem* human tissue, their identification approach relied on the analysis of protein-coding genes expressed in the CNS of adolescent mice. In this context, hMO scRNAseq datasets (153) may thus constitute more relevant tools to explore the cell types (and subtypes) involved in PD pathophysiology. Single-cell approaches may also constitute an ideal readout to assess the molecular effects of somatic mosaicism (such as *SNCA* CNVs), which can be induced in organoids through the use of transfection and mixing approaches (154).

Furthermore, considering that PD SNPs mainly fall into non-coding regions of the genome (32), combining readout modalities such as RNAseq, ChIP-seq, ATAC-seq, and proteomics can help dissect complex molecular networks by including non-coding elements and epigenetic modifications. For example, Inoue et al. (155) used a combination of multiple modalities including lentivirus-based massively parallel reporter assay to identify key regulatory elements and dynamics involved in the neural induction of embryonic stem cells. They also found a significant enrichment of neurological disorder GWAS variants in regions with H3K27ac histone modifications. A similar approach was also recently applied to forebrain organoid models (126). The authors used a combination of RNA-seq and ATAC-seq to map changes in gene expression, chromatin accessibility, and transcription factor dynamics in purified neuronal and glial lineages over 20 months of differentiation. They then also incorporated GWAS risk gene mapping to identify specific cell types and neurodevelopment stages involved in ASD and schizophrenia. Multimodal -OMICs integration has also proven to be a useful strategy to identify the repertoires of long non-coding RNAs (lncRNA) in mDA neurons (41), in which GWAS SNP mapping identified 8 lncRNA possibly involved in PD pathophysiology. With the development of single-cell approaches [see recent reviews (156, 157)], identification of cell subtypes involved in PD pathophysiology may further increase our understanding of the disease.

CRIPSR-based technology may also be of particular use to explore the molecular networks involved in PD, notably through the use of genetic perturbation screens, and through enhanced disease modeling [see review (158)]. Indeed, Crispr-based techniques offers an unprecedented method to model the polygenic liability of complex disorders such as PD *in vitro*. In a proof of concept experiment, Schrode et al. (159) used Crispr-based allelic conversion and activation/inhibition to manipulate four risk genes associated with schizophrenia in hiPSC-derived neuronal cultures, and demonstrated a synergistic effect on synaptic function. Furthermore, combining such approaches with the use of environmental stressors associated with PD may constitute a unique opportunity to model gene ^*^ environment interactions *in vitro*. For instance, while several studies using hMO and related 3D cultures have shown mDA neuronal vulnerability to acute treatment with mitochondrial toxins such as rotenone, MPTP, and 6-OHDA (67, 77, 83, 85), studying the interaction of lower to medium risk genetic variants with chronic, low-dose environmental stressors may allow us to reproduce idiopathic trajectories of PD in a dish.

### Therapeutic Opportunities

Organoids also constitute a relevant platform to identify novel therapeutic compounds and to assess their efficacy on specific phenotypes. Kim et al. (77) showed that alpha-synuclein accumulation could be reduced in *LRRK2* G2019S hMO through treatment with a LRRK2 kinase activity inhibitor (GSK2578215A), but also by knocking down the expression of *TXNIP*, which their study had identified as a central mediator of G2019S pathology. Jarazo et al. (80) also found that treatment with the HP-ß-CD compound improved mDA neuronal differentiation in *PINK1* and *PRKN*-mutated hMO, likely through increased mitophagy.

A few elements should be heeded regarding therapeutic developments using hMO. First of all, as organoid generation is prone to variability, taking measures to reduce this confounding factor (detailed in section Addressing variability in organoid differentiations) is essential to accurately assess the potential of therapeutic targets. Secondly, the organoids generated should be extensively characterized, in order to best plan the modalities of therapeutic testing and to help identify the appropriate readouts to quantify the effects. In this sense, disease-modifying treatments targeting deficits in early differentiation of mDA neurons may require different modalities and readouts than those aiming at increasing the survival of compromised, mature cells.

In the long run, hMO technology opens up perspectives for personalized medicine. The study by Ahfeldt et al. (74) identified at least two distinct molecular phenotypes in hMO derived from either *PRKN*^−/−^, or *ATP13A2*/*DJ1* mutated lines, indicating that familial PD mutations induce different pathological cascades, which may call for different therapeutic strategies. Furthermore, personalized medicine approaches may also be explored in cases of non-familial PD. An initial experiment for example proved that cellular alteration in hiPSC-derived neurons from patients with Bipolar Disorder were reversed by lithium treatment only if the patients were also responsive to the medication (160). More recently, Lang et al. (161) proved that mDA neurons from patients carrying an identical variation in a common risk gene (*GBA* N370S) could be stratified based on their molecular profile using RNA sequencing. Clinical follow-up confirmed that their strategy had indeed isolated a patient who proved to be non-responsive to levodopa treatment, and who received a revised diagnosis of progressive supra nuclear palsy. They also identified a causative role of the mis-localization of a class IIa histone deacetylase (HDAC4) in the remaining cell lines, which was then also observed in 2 out of 4 idiopathic PD-derived mDA neuron lines. As modulating the activity or localization of HDAC4 alleviated the cellular PD phenotype, this study suggests that deriving personalized medicine approaches from hiPSC-derived cultures may indeed be a reality in the foreseeable future.

Finally, transplantation of hMO into PD patients' brains to compensate for their loss of mDA neurons also constitutes a promising therapeutic endeavor. Recent studies have demonstrated that stem cell-derived mDA neurons or mDA progenitors could indeed functionally integrate into striatonigral circuits (162), and provide some symptomatic relief in a non-human primate model of PD without forming tumors (163). As organoids have been shown to efficiently integrate into rodent neural circuits after transplantation (100), using dopamine-producing hMO may prove to be a useful development for therapeutic purposes. In this regard, a recent patent (115) indicates that hMO transplantation in a unilateral 6OHDA mouse model of PD could reduce turning behavior in response to an apomorphine challenge, suggesting that the hMO may have functionally integrated into the host organism.

## Conclusion

Since their first description in 2014, hMO have proven to efficiently generate functional, NM-producing mDA neurons with A9/A10-like identity in structures that recapitulate features of composition and organization of the human midbrain. PD modeling studies using hMO have also shown their ability to reproduce elements of the disease, such as α-syn accumulation and impairment of mitochondrial function. Interestingly, some mutations did not elicit such phenotypes, suggesting that hMO may also be suited to investigate PD heterogeneity. Whether the observed phenotypes are due to developmental or aging-related pathological mechanisms remains however unclear, as limitations inherent to hiPSC-derived organoid cultures might for now prevent the dissociation of such aspects. Nevertheless, recent studies suggest that these limitations can be overcome through optimisation of culture systems, “aging” strategies and transplantation into host organisms. Future development of hMO co-culture systems will also help study neuroinflammatory processes and interactions with other brain areas involved in PD pathophysiology. Combined with genetic engineering and multimodal molecular readouts, hMO may thus provide a crucial platform to explore the molecular basis of PD, with direct therapeutic implications.

## Author Contributions

BG and HC wrote the initial draft. BG, HC, and PR reviewed and edited the final manuscript. All authors contributed to the article and approved the submitted version.

## Conflict of Interest

The authors declare that the research was conducted in the absence of any commercial or financial relationships that could be construed as a potential conflict of interest.
